# Genotoxicity induced by iodine-131 in human cultured lymphocytes

**DOI:** 10.2478/intox-2013-0013

**Published:** 2013-06

**Authors:** Seyed Jalal Hosseinimehr, Nayereh Shafaghati, Monireh Hedayati

**Affiliations:** Department of Radiopharmacy, Faculty of Pharmacy, Mazandaran University of Medical Sciences, Sari, Iran

**Keywords:** iodine-131, radioiodine, genotoxicity, micronucleus, lymphocyte

## Abstract

131-radioiodine has been widely used as an effective radionuclide for treatment of patients with thyroid diseases. The purpose of this study was to investigate the genotoxic effects of iodine-131 in human cultured lymphocytes. Whole blood samples from human volunteers were incubated with iodine-131 (10, 50, 100 µCi/1.5ml) for 2 h. The lymphocytes were mitogenically stimulated to allow for evaluation of the number of micronuclei in cytokinesis-blocked binucleated cells. At the dose 100 µCi, iodine-131 induced genotoxicity by an 8.5 fold increase in the frequency of micronuclei in human lymphocytes compared with the control group.

## Introduction

Over the past 60 years in nuclear medicine, iodine-131 has been widely used for treatment of patients with thyroid diseases like hyperthyroidism and thyroid cancer. iodine-131 has a physical half life of 8.02 days, it emits a beta particle with a high energy of 0.61 MeV and medium energy of 0.20 MeV and gamma ray of 0.36 MeV (Robbins & Schlumberger, 2005). After administration of iodide, it is secreted into saliva and its salivary concentration has been reported to vary from 20 to 100 times higher compared to that found in serum. Beta minus produced by iodine-131 can destroy cells with direct or indirect effects. Ionizing radiation produces reactive oxygen species (ROS); these toxic products can interact with critical macromolecules to induce cellular damage (Hosseinimehr, 2010). ROS can attack critical macromolecules such as DNA resulting in DNA damage, chromosomal breaks and cell death at high radiation dose (Little, 2000). Although, iodine-131 damages by with these mechanisms tumor cells, it can induce side effects on normal tissue due to unwanted accumulation of this radionuclide in healthy organs. Short and long term side effects related to radioiodine therapy include nausea, sialadenitis and hematological depression. In addition,there is an induction of secondary cancer and genetic damage following iodine-131 therapy. An increased a risk of leukemia, bladder cancer and colorectal cancer were reported after iodine-131 therapy in patients (de Vathaire *et al.*, 1997; Edmonds & Smith, 1986; Grudeva-Popova *et al.*, 2007; Kolade *et al.*, 2005; Schroeder *et al.*, 2012). There are several studies showing that genetic damage was increased in patients after iodine-131 therapy. The cytokinesis-blocked micronucleus assay is a well-established cytogenetic method for measuring chromosomal DNA damage in cultured human lymphocytes from humans exposed to genotoxic agents. Micronuclei originate from chromosome breaks and fragments at anaphase during mitosis (Fenech, 2000). *In vivo*, there are several studies reporting increased percentage of micronuclei in lymphocytes of patients during iodine-131 therapy (Gutierrez *et al.*, 1997; Watanabe *et al.*, 2004). There are few papers about radioiodine toxicity and doses of iodine-131 inducing genetic damage to human lymphocytes *in vitro*. These *in vitro* established and setup experiments would help assess radioprotection in iodine-131 toxicity. The aim of this study was to set up and determine the genotoxicity induced by iodine-131 at different doses on human peripheral blood lymphocyte cells *in vitro*.

## Material and methods

### Irradiation protocol

The study protocol was approved by the Ethical Committee of the University. After obtaining written informed consent, twelve milliliter blood samples were collected in heparinized tubes from three healthy, nonsmoking male volunteers, aged 25–35 years. ^131^INa in sterile solution was prepared by EOI, Tehran, Iran, and was used freshly. The blood was divided into four 1.5-ml tubes, one for each of the six study groups: control, iodine-131 at doses 10, 50 and 100 µCi/1.5 mL (final concentrations). Blood samples were incubated with iodine-131 at 37 °C for 2 h. After incubation, RPMI 1640 medium was added to each tube and the cultures were centrifuged at 1500 g for 8 minutes. To separate iodine-131 from the whole blood, the upper (less dense) solution was removed and blood was transferred for micronucleus assay.

### Micronucleus assay

Of each sample (control and irradiated groups) 0.5 mL was added to 4.5 mL of RPMI 1640 culture medium (Gibco, USA), which contained 10% fetal calf serum, 2 mM glutamine (Sigma, USA), 0.1 mL/5mL phytohemagglutinin (Gibco, USA) and antibiotics (Penicillin 100 IU/ml, Streptomycin 100 µg/ml) (Gibco, USA). All cultures were set up in duplicate and incubated at 37±1 °C in a humidified atmosphere of 5% CO_2_/95% air. Cytochalasin B (Sigma, final concentration: 6 µg/ ml) was added after 44 hours of culture incubation. At the end of 72 h of incubation, the cells were collected by centrifugation. Cells were treated with a fixative solution three times (methanol:acetic acid). Fixed cells were stained with Giemsa (Merck, Germany) solution (20%). All slides were coded and evaluated at 100× magnification for the micronuclei frequency in binucleated cells with well-preserved cytoplasm. To be scored as micronuclei, candidates had to have a diameter between 1/16^th^ and 1/3^rd^ of main nuclei, be non-refractile, and not linked to or overlap with the main nuclei (Fenech, 2000). At each blood collection end point, at least 1000 binucleated cells from duplicate irradiated and control cultures from each volunteer were examined to record the frequency of micronuclei.

### Statistical analysis

At each blood collection, the prevalence of micronuclei was recorded for each volunteer. The data were analyzed using ANOVA with Tukey's HSD posthoc test.

## Results

**Figure 1:**
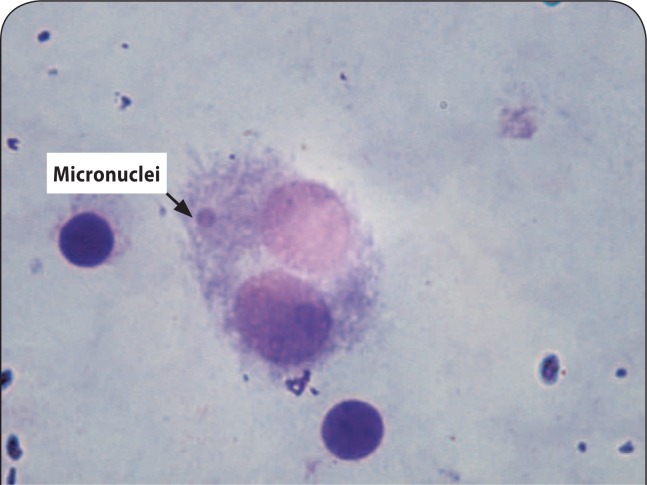
A typical binucleated lymphocyte with micronucleus in our study.

The percentage of micronuclei in the lymphocytes of volunteers treated with 10, 50 and 100 µCi of iodine-131 was 1.23 ±0.15, 1±0.2 and 4.83±0.9, respectively, while it was 0.56 ±0.35 in non-treated control lymphocytes (Table 1). The frequency of micronuclei (an indication of the genotoxic effects of internal exposure irradiation) after pre-incubation with iodine-131 at the dose of 100 µCi was significantly higher than in the control group (*p<*0.001). A statistically significant difference was observed between control and the dose of 10 µCi of iodine-131 (*p<*0.05) in increasing micronuclei in lymphocytes. No statistically significant difference was observed between control and 50 µCi of iodine-131. Total micronuclei values were 2.1, 1.7 and 8.5 fold higher in the samples treated with iodine-131 at concentrations of 10, 50 and 100 µCi, respectively, than in controls (Table 1). A typical picture of a binucleated cell with micronuclei found in this study is shown in Figure 1.

**Table 1:** Percentages of micronuclei induced *in vitro* by different activity (µCi) of iodine-131 in cultured blood lymphocytes from human volunteers.

Volunteer No.	Control	10	50	100
Volunteer 1	0.9	1.1	0.8	4
Volunteer 2	0.2	1.2	1.2	4.7
Volunteer 3	0.6	1.4	1	5.8
Mean ± SD	0.57±0.35	1.2±0.15* †	1±0.2‡	4.8±0.9***

# 1000 BN cells were examined in each culture.

* *p<*0.05 statistically significant compared to control

† not significant compared to dose of 50 µCi

‡ not significant between doses 50 µCi and control

*** *p<*0.001 statistically significant compared to control

## Discussion

In this study, we observed genotoxic effects induced by iodine-131 presented by increased umber of micronuclei in lymphocytes at the dose of 100 µCi. Monterio Gil investigated DNA damage, namely micronuclei in peripheral lymphocytes, in thyroid cancer patients after iodine-131 therapy. The number of micronuclei in the cells increased after one month of treatment (Monteiro Gil *et al.*, 2000). Ballardin *et al.* (2002) observed 4-fold increase in the frequency of micronuclei 7 days after radioiodion therapy in patients. The frequency of micronuclei declined slowly and reached the baseline 180 days after therapy (Ballardin *et al.*, 2002). Other studies reported an increasing frequency of micronuclei in patients after iodine-131 therapy (Gutierrez *et al.*, 1997; Watanabe *et al.*, 2004). The micronuclei assay can be used as a valuable endpoint and sensitive method for studying radiation biology for assessment of genetic damage. Although, iodine-131 is considered a very useful radionuclide in reducing thyroid activity, as a genotoxic agent it may produce secondary cancer incidence in patients (Fallahi *et al.*, 2011; Iyer *et al.*, 2011; Sawka *et al.*, 2009). In patients after iodine therapy, the incidence of leukemia was significantly increased and found to be more frequent than other cancers (Sawka *et al.*, 2009). Our study showed a significant increase of micronuclei in lymphocytes treated with iodine-131 at the dose 100 µCi. iodine-131 emits gamma and beta rays. The latter has a short range board with higher destroying effects on cells as compared to gamma rays. Induction of oxidative stress is one of the main mechanisms operative in the therapeutic and/or side effects of iodine-131. Oxidative stress may be related to DNA damage (Monteiro Gil *et al.*, 2000). In the literature, we have not found any information about serum concentration and pharmacokinetics of iodine-131 in patients after iodine-131 therapy. If it was determined, it would help us to find a connection between *in vivo* and *in vitro* doses. This study showed that determination of micronuclei in lymphocytes is a suitable and easy method for assessment of genotoxicity of iodine-131 *in vitro* in cultured lymphocytes.
